# Endovascular thrombectomy versus intravenous thrombolysis for primary distal, medium vessel occlusion in acute ischemic stroke

**DOI:** 10.1515/med-2024-0966

**Published:** 2024-05-13

**Authors:** Giancarlo Salsano, Antonio Salsano, Bruno Del Sette, Alessio D’Alonzo, Davide Sassos, Andrea Alexandre, Alessandro Pedicelli, Riccardo Di Iorio, Francesca Colò, Lucio Castellan

**Affiliations:** Department of Radiology and Interventional Radiology, IRCCS Ospedale Policlinico San Martino, Genoa, Italy; DISC Department, University of Genova, Genoa, Italy; Clinica Neurologica IRCCS Azienda Ospedaliera Universitaria San Martino, IST, Genoa, Italy; UOSA Neuroradiology Unit, Department of Radiology, Radiotherapy and Oncology and Hematology, Fondazione Policlinico Universitario A. Gemelli IRCCS, Rome, Italy; Neurology Unit, Department of Aging, Neurological, Orthopedic and Head and Neck Sciences, Fondazione Policlinico Universitario A. Gemelli IRCCS, Rome, Italy; Department of Neurosciences, Catholic University School of Medicine, Rome, Italy

**Keywords:** DMVO, endovascular therapy, IVT, acute ischemic stroke

## Abstract

**Introduction:**

In the absence of clinical trials, the benefit of endovascular therapy (EVT) on the treatment of acute ischemic stroke (AIS) with primary distal and medium vessel occlusions (DMVO) is still not well defined. The aim of the study is to evaluate EVT with or without intravenous thrombolysis (EVT ± IVT) in primary DMVO stroke in comparison with a control cohort treated with IVT alone.

**Methods:**

We analysed all consecutive AIS with proven primary DMVO. Primary endpoints were excellent outcome, functional independence at 3 months follow-up, and early neurologic improvement at 1 day after treatment.

**Results:**

One hundred and fourteen patients with DMVO strokes were included between 2019 and 2023. Propensity-weighted analysis showed no significant differences in EVT ± IVT vs IVT for the excellent outcome (adjusted OR [aOR], 1.575; 95% CI, 0.706–3.513), functional independence (aOR, 2.024; 95% CI, 0.845–4.848), early neurological improvement (aOR, 2.218; 95% CI, 0.937–5.247), mortality (aOR, 0.498; 95% CI, 0.177–1.406), symptomatic intracranial haemorrhage (aOR, 0.493; 95% CI, 0.102–2.385), and subarachnoid haemorrhage (aOR, 0.560; 95% CI, 0.143–2.187). The type of revascularization did not influence the percentage of cerebral volume lost (adjusted linear regression estimate, −19.171, *t* value, 11.562; *p* = 0.104).

**Conclusions:**

This study supports the hypothesis that patients with primary DMVO stroke treated with EVT (±IVT) or IVT alone have comparable outcomes.

## 1 Introduction

Since the publication of randomized controlled trials (rCTs) focusing on mechanical thrombectomy for acute ischemic stroke (AIS) with large vessel occlusions (LVO) of the anterior circulation [1,2,3,4,5,6,7], treatment indications have been dramatically expanded. Trials established the overwhelming superiority of endovascular therapy (EVT) with or without intravenous thrombolysis (IVT) over best medical care. In particular, mechanical thrombectomy provided higher rates of functional independence (score of 0–2 on the modified Rankin scale [mRs]) and excellent outcome (score of 0–1 on mRs) than medical therapy, reducing mortality rates in patients with the following criteria: (1) occlusion of the internal carotid artery or/and the M1 segment of the middle cerebral artery (MCA); (2) baseline National Institute of Health Stroke Scale (NIHSS) score ≥6; and (3) Alberta Stroke Program Early Computed Tomographic Score (ASPECTS) ≥6.

Patients with ischemic stroke due to M2 segment MCA occlusions were either deliberately excluded (e.g. in the ESCAPE, SWIFT PRIME, REVASCAT, THRACE, and PISTE trials) or were undersampled (e.g. in the MR CLEAN and the EXTEND IA trials) in rCTs. Meta-analyses [8,9,10] of multicentre prospective cohorts and randomized clinical trials confirmed the potential benefit of EVT for dominant or codominant branches of the M2 segment of the MCA occlusion over best medical treatment (BMT).

Moreover, benefits of EVT were also found in patients with stroke onset beyond 6 h [11,12], in elderly patients/octogenerians [13,14], low ASPECT score values [15,16], and basilar artery occlusions [17].

In distal and medium vessel occlusions (DMVO), it is difficult to weigh the risk–benefit ratio of the endovascular approach due to small at-risk tissue volumes, lower NIHSS scores, and higher risk of procedure-related complications. Although DMVO tends to be less devastating than LVO, an eloquent peripheral brain vessel occluded may substantially degrade functional outcomes and quality of life. However, pieces of evidence of good outcomes after EVT are limited to the M2 segment, while there are no specific recommendations by international guidelines for distal occlusion sites [18].

The safety and efficacy of EVT in primary DMVO, including distal or nondominant M2 segment and M3 segment of the MCA, distal segments of the anterior cerebral artery (ACA), and posterior cerebral artery (PCA), is still a matter of debate.

### 1.1 Aim

The aim of the study is to evaluate EVT ± IVT in primary DMVO stroke in comparison with a control cohort treated with IVT alone.

## 2 Materials and methods

### 2.1 Study design, participants, and procedures

We conducted a retrospective analysis of prospectively collected data in institutional databases of two Italian centres (Table S1). We analyse all consecutive AIS with primary DMVO on initial cerebral computed tomography angiogram (CTA) that received IVT or EVT, either alone or in combination with IVT, within 16 h from the last known normal clinical status. Patients’ selection for EVT ± IVT or IVT group was at the discretion of the treating physicians. Both anterior and posterior circulation distal occlusions were defined according to the international consensus statement of Saver et al. [19].

We prospectively collected baseline demographic characteristics, vascular risk factors (diabetes, smoking, hyperlipidaemia, hypertension, obesity), and other comorbidities (atrial fibrillation, neoplasia, previous stroke, chronic kidney disease, in-hospital stroke, antiplatelet and home anticoagulant therapy). NIHSS scores were evaluated by stroke neurologists at baseline, at day 1, and at discharge. Early ischemic changes were measured by the ASPECTS on noncontrast-enhanced CT scan. The site of distal cerebral occlusion was assessed by an experienced neuro-radiologist on CT angiography and confirmed by digital subtraction angiography when performed. The volume of cerebral tissue at risk of infarction (CTP_TMAX>6s_) was automatically calculated using pre-treatment CT perfusion imaging with RAPID AI software (Rapid AI, San Mateo, CA, USA) [20].

Door-to-needle time (from symptom onset or last known well for patients with wake-up strokes), onset to groin time, onset to recanalization time, and procedure time were recorded.

Endovascular reperfusion grade was assessed with the eTICI score [21]; successful reperfusion was defined as a modified score of 2b or higher. Endovascular procedure-related complications were reported (subarachnoid haemorrhage [SAH]; embolization to new arterial territory/distal embolization in target territory; arterial dissection). Early neurologic improvement was evaluated with NIHSS at 1 day after treatment.

Functional independence and excellent outcome at 3 months follow-up were assessed on mRS. Final infarction volume (FIV) was calculated as the total hypodense volumes in each axial slice ipsilateral to DMVO using segmentation CT software [22]. Oedema-producing sulcal effacement and haemorrhagic transformation were not excluded in the FIV. Symptomatic intracerebral haemorrhage (sICH) was defined according to ECASS-2 criteria [23].

### 2.2 Inclusion and exclusion criteria, endpoints, and outcome definitions

#### 2.2.1 Inclusion and exclusion criteria

We enrolled all patients affected by AIS with demonstrated primary DMVO on initial CT angiography treated with IVT or EVT ± IVT.

Patients with DMVO of both anterior (distal or nondominant M2-segment, M3 and M4-segment of MCA, A2 and A3-segment of ACA) and posterior circulation (P2 and P3-segment PCA), without restriction for NIHSS, age ≥18 years and with 3-month mRS score, were included. Revascularization techniques were at the discretion of interventional neuroradiologists depending on a specific case.

Patients with LVOs, dominant M2-segment occlusions, secondary DMVO, and tandem lesions were excluded.

#### 2.2.2 Primary and secondary endpoints

Primary endpoints were the following: excellent outcome and functional independence at 3 months follow-up; early neurologic improvement at 1 day after treatment.

Secondary endpoints were the following: endovascular procedure-related complication (arterial dissection, SAH or arterial perforation, and embolization to new arterial territory/distal embolization in target territory), symptomatic intracranial haemorrhage, mortality and impact of treatment on reducing the percentage of cerebral volume lost.

Outcomes and DMVO anatomy definitions are provided in the Supplementary material.

### 2.3 Statistical analysis

Categorical data were presented as frequencies and percentages and compared using the Chi-square test or Fisher’s exact test where appropriate. Continuous variables were expressed as median and interquartile range (IQR) and compared using a two-tailed Mann–Whitney test for non-parametric distributions. To adjust for confounding, a doubly robust method (a combination regression model with inverse probability treatment weighting [IPTW] by propensity score) was used to estimate the causal effect of the exposure on the outcomes [24]. For this purpose, a propensity score was developed to minimize the differences between patients undergoing intravenous rtPA vs EVT [25]. The covariates were gender, age, diabetes, dyslipidaemia, coronary artery disease, hypertension, smoke, cervical internal carotid artery stenosis, atherosclerotic cause of vessel obstruction, and baseline NIHSS score. Using the estimated propensity scores as weights, an inverse probability weighting model was used to generate a weighted cohort [26]. C-statistics were calculated to ascertain the validity of the propensity score.

Sensitivity analyses excluding patients with multiple distal occlusions and predictors of excellent outcomes were applied. Variables significantly associated (*p* < 0.05) with excellent outcomes at univariate analysis were included in a parsimonious multivariable stepwise logistic regression model with selection based on the Akaike information criterion using Firth’s bias reduction method. Results were reported as odds ratio (OR), 95% confidence limits (95% CI), and *p*-value. The final model was internally validated using 1,000 bootstrapping iterations. The receiver operating characteristic (ROC) curve was used to estimate the discrimination power. Calibration was assessed through the Hosmer–Lemeshow test.

Statistical analyses were performed using R software (version 4.2.1; R Foundation for Statistical Computing, Vienna, Austria).

## 3 Results

### 3.1 Baseline

**Figure 1:**
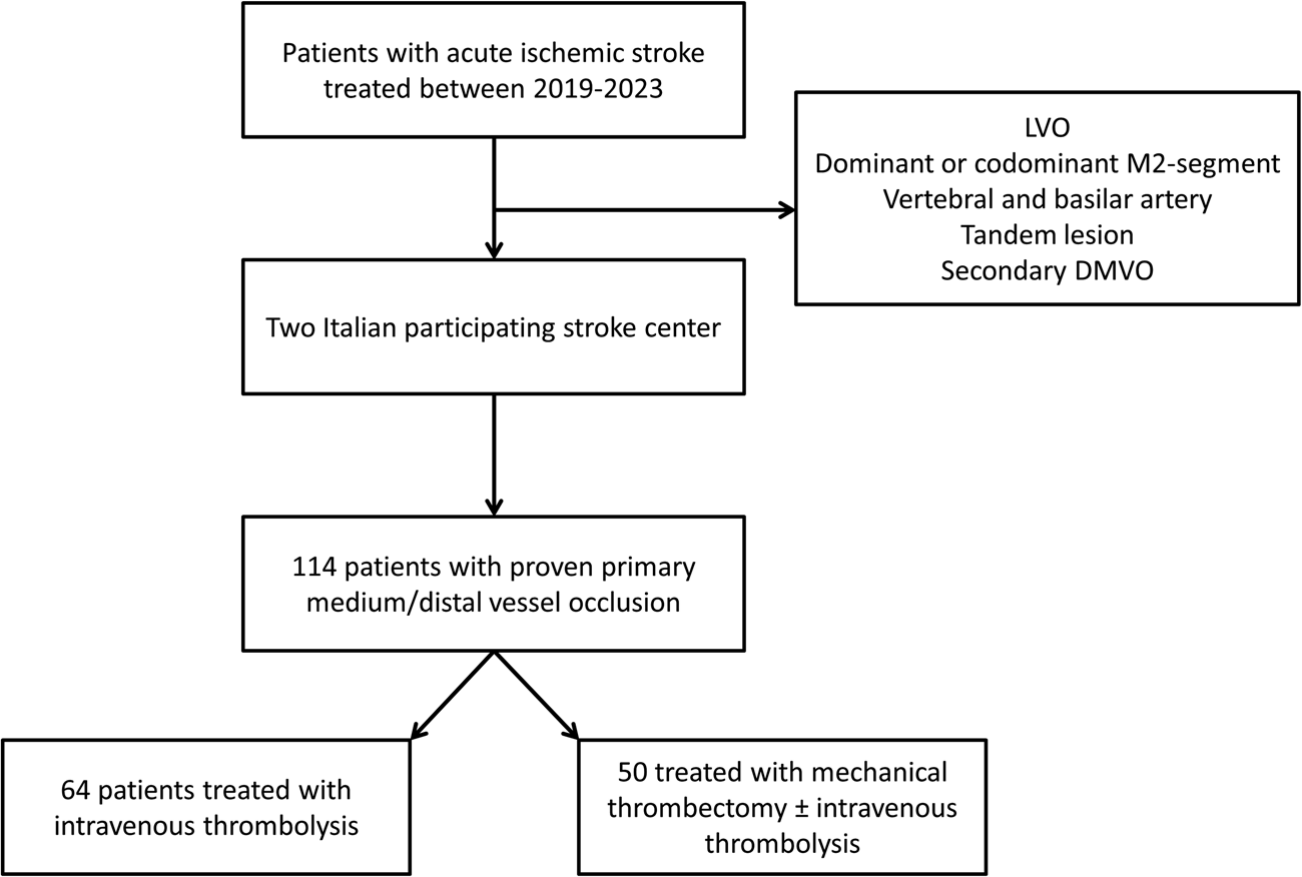
Study flow chart.

A total of 114 consecutive patients with imaging-proven DMVO were included between January 2019 and January 2023 (Figure 1). Fifty were treated with EVT ± IVT ([median] age, 78 [IQR: 72.00–83.75] years; 32 men [64%] and 18 women [36%]) and the remaining 64 received IVT alone ([median] age, 80 [IQR: 74.00–84.25] years; 29 men [45.3%] and 35 women [54.7%]). The baseline characteristics and medical history between the two groups of treatment are summarized in Table 1. About 34% of the EVT patients received intravenous tPA. The baseline NIHSS score (mean [SD], 11.02 [4.92] vs 8.44 [5.40]; *p* = 0.01) and home anticoagulant therapy (mean [SD], 10 [20.0] vs 1 [1.6]; *p* = 0.03) were significantly higher in the EVT group compared with the IVT group. On pre-treatment CT angiogram, 104 patients (91.2%) had only one distal vessel occlusion, and the remaining 10 patients (8.8%) presented with multiple distal vessel occlusion.

**Table 1:** Baseline characteristics and medical history

Variable	IVT group (64)	EVT ± IVT group (50)	*p* value
Female, no. (%)	35 (54.7)	18 (36.0)	0.073
Age, Y, median (IQR)	80.00 [74.00, 84.25]	78.00 [72.00, 83.75]	0.583
Weight, kg, median (IQR)	70.00 [65.00, 80.00]	72.00 [65.25, 78.00]	0.618
Atrial fibrillation, no. (%)	16 (25.0)	17 (34.0)	0.399
Intrahospital stroke, no. (%)	1 (1.6)	2 (4.0)	0.828
Diabetes, no. (%)	13 (20.3)	8 (16.0)	0.729
Dyslipidemia, no. (%)	17 (26.6)	12 (24.0)	0.924
CAD, no. (%)	16 (25.0)	13 (26.0)	1.000
Cervical internal carotid artery stenosis, no. (%)	8 (12.5)	9 (18.0)	0.580
COPD, no. (%)	4 (6.2)	2 (4.0)	0.911
Previous stroke, no. (%)	7 (10.9)	10 (20.0)	0.279
Neoplasia, no. (%)	1 (1.6)	3 (6.0)	0.444
Dementia, no. (%)	1 (1.6)	1 (2.0)	1.000
Hypertension, no. (%)	40 (62.5)	31 (62.0)	1.000
Smoking habit, no. (%)	6 (9.4)	6 (12.0)	0.884
Chronic kidney disease, no. (%)	3 (4.7)	2 (4.0)	1.000
Home antiplatelet therapy, no. (%)	29 (45.3)	18 (36.0)	0.418
Home anticoagulant therapy, no. (%)	1 (1.6)	10 (20.0)	0.003
Statins, no. (%)	10 (15.6)	10 (20.0)	0.718
**mRS pre stroke, no. (%)**			0.914
0	52 (81.2)	42 (84.0)	
1	7 (10.9)	5 (10.0)	
2	5 (7.8)	3 (6.0)	
**Occlusion side, no. (%)**			0.258
Right	30 (46.9)	24 (48.0)	
Left	34 (53.1)	24 (48.0)	
Bilateral	0 (0.0)	2 (4.0)	
Atherosclerotic occlusion, no. (%)	25 (39.1)	13 (26.0)	0.205
Tissue at risk of infarction (CTP_tmax_ > 6 s), ml, mean (SD)	50.08 (39.39)	98.10 (107.72)	0.134
Occlusion site on CTA, no. (%)			0.186
Distal or non codominant M2-segment	19 (27.5)	26 (47.3)	
M3-segment	32 (46.4)	21 (38.2)	
M4-segment	1 (1.5)	0 (0)	
A2/A3-segment		6 (8.7)	6 (10.9)
P2/P3-segment	11 (15.9)	2 (3.6)	
Multiple distal occlusion	5 (7.2)	5 (9.0)	
**Menon score, no. (%)**			0.075
0	1 (1.6)	4 (8.0)	
1	1 (1.6)	0 (0.0)	
2	5 (7.8)	11 (22.0)	
3	15 (23.4)	12 (24.0)	
4	21 (32.8)	9 (18.0)	
5	21 (32.8)	14 (28.0)	
Intravenous tPA, no. (%)	64 (100.0)	34 (68.0)	<0.001
Baseline NIHSS score (mean (SD))	8.44 (5.40)	11.02 (4.92)	0.010
Time from onset to IVT, min, (mean (SD))	109.33 (70.88)	161.19 (93.95)	0.003
Time from onset to groin puncture, min (mean (SD))	NA	243.20 (134.09)	NA
Time from onset to recanalization, min (mean (SD))	NA	287.67 (131.19)	NA
Procedure time, min (mean (SD))	NA	47.10 (33.45)	NA

CAD: coronary artery disease; COPD: chronic obstructive pulmonary disease; Y: year; kg: kilogram; ml: millilitres; min: minutes; IQR: interquartile range; SD: standard deviation; CTA: computed tomography angiogram; EVT, endovascular therapy; IVT, intravenous tPA; NA: not applicable.

The mean time from onset to intravenous tPA administration was significantly higher in the EVT group in comparison with the IVT group alone (mean [SD], 161.19 min [93.95] vs 109.33 min [70.88]; *p* = 0.03).

**Figure 2:**
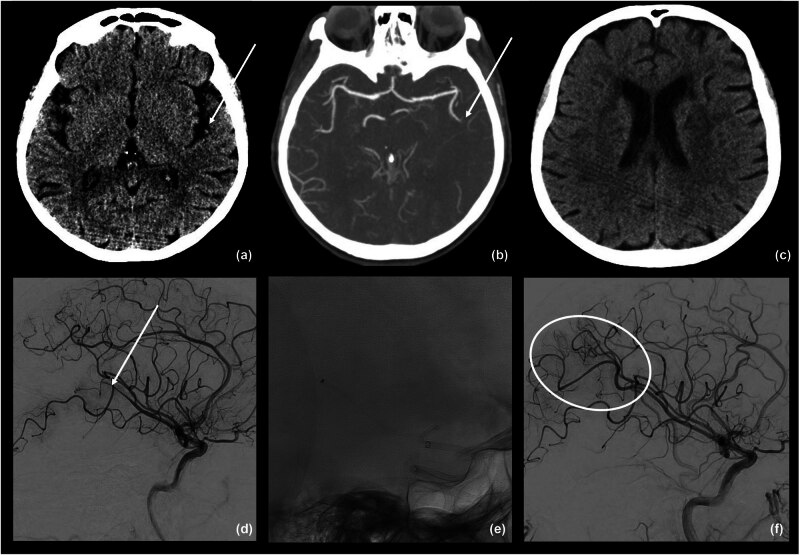
An illustrative case of 67-year-old woman with AIS and severe aphasia (7-point NIHSS). Non-contrast-CT scam shows focal hyperdensity of M3-segment of left MCA (a) suspected for arterial thrombosis. This finding is confirmed by CT angiography (b) and digital subtraction angiography (d), which show the occlusion of the arterial branch that supplies the Wernicke area (white arrows). After a single thrombectomy with 3 Max aspiration device (e), arterial recanalization (TICI 3) was achieved (f). Follow-up CT scan does not identify ischemic changes (c).

The mean procedure time, from the first angiogram to the final control angiogram, was 47 (16–180) min. The first-line revascularization technique was contact aspiration in 62% and stent retriever (with or without aspiration) in 38%. A single pass of revascularization device was performed in 54%, 2 passes in 32%, and 3 or more in 14%. Successful recanalization (TICI score ≥2b) was achieved in 45 (90%) patients, and complete recanalization (TICI score = 3) in 23 (46%) patients (Figure 2). Four patients had a procedure-related complication (SAHs). Neither distal embolization to new arterial territory/distal embolization in target territory nor arterial dissection was recorded.

### 3.2 Clinical outcomes

**Figure 3:**
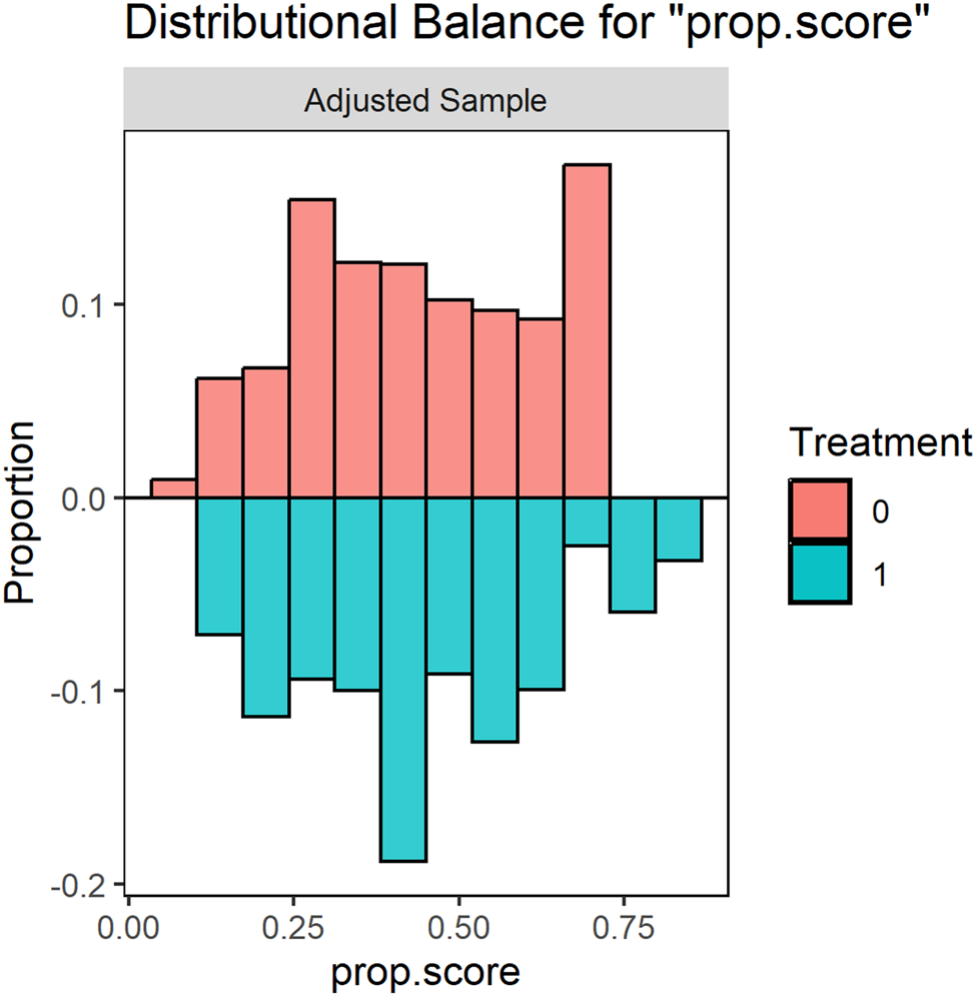
Balance plot. Distributional balance for propensity score. Red: adjusted sample; blue: unadjusted sample; 0 = mechanical thrombectomy; 1 = IVT.

**Figure 4:**
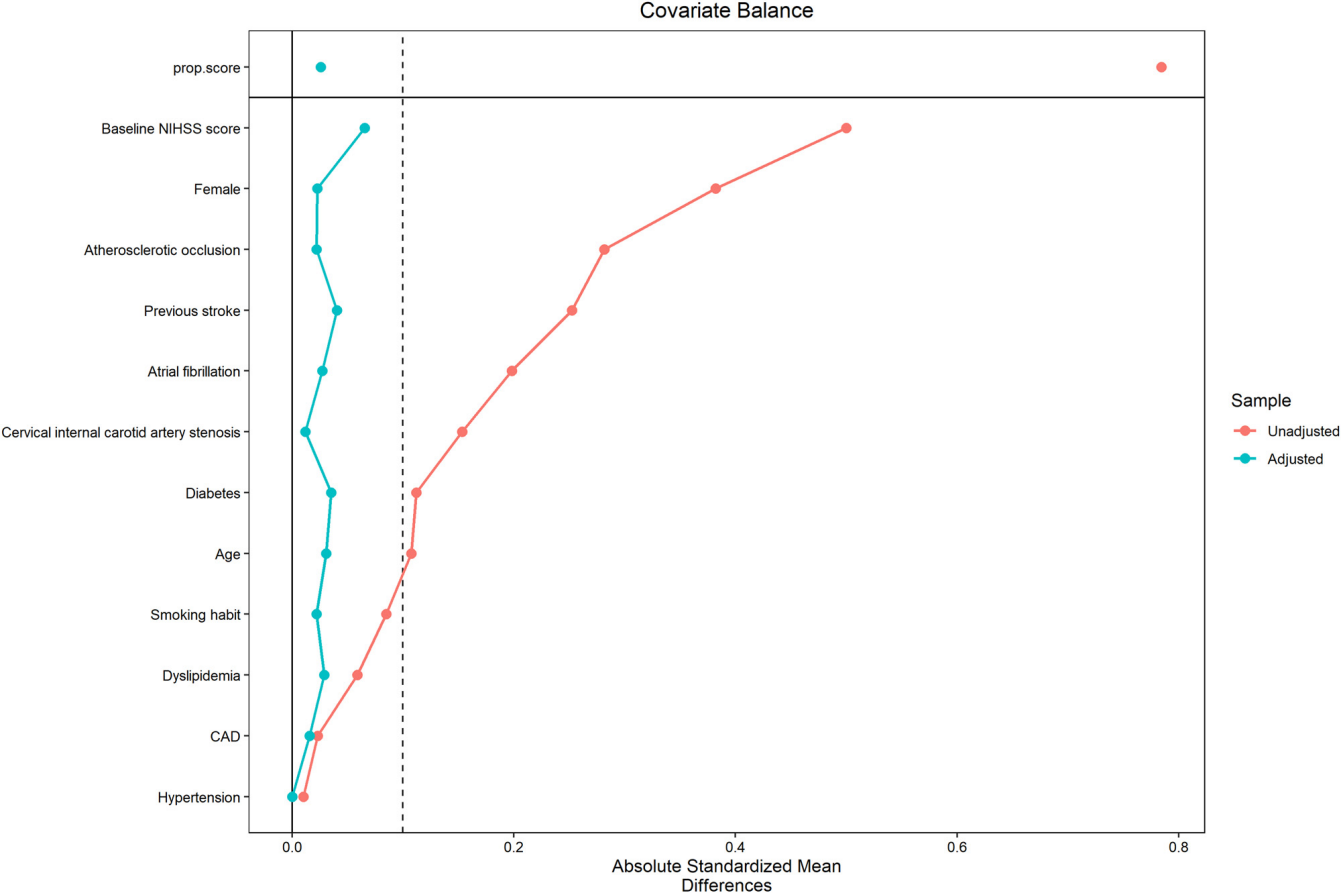
The love plot shows the distribution of covariates before (red) and after (blue) IPTW. IPTW: inverse probability of treatment weighting.

Table 2 reports clinical outcomes before (crude rates) and after doubly robust adjustment. As shown in Table S1 and Figures 3 and 4, all the covariates of the weighted cohorts were well balanced between the EVT ± IVT group and the IVT alone group. The C-statistics of the propensity score was 0.710 (Figure S1).

**Table 2:** Clinical outcomes and the doubly robust matching estimators for confounding adjustment for intravenous tPA vs EVT

Variables	Overall series			Doubly robust adjustment§		
	IVT *N* = 64 pts	EVT ± IVT *N* = 50 pts	*p* value	OR	95% CI	*p* value
Excellent outcome (mRS = 0–1) at 3 months (%)	36 (56.2)	27 (54.0)	0.960	1.575	0.706–3.513	0.269
Functional independence (mRS = 0–2) at 3 months (%)	42 (65.6)	37 (74.0)	0.449	2.024	0.845–4.848	0.116
Early neurological improvement (%)	17 (26.6)	24 (48.0)	0.030	2.218	0.937–5.247	0.073
Mortality (%)	13 (20.3)	8 (16.0)	0.729	0.498	0.177–1.406	0.191
sICH (%)	6 (9.4)	3 (6.0)	0.754	0.493	0.102–2.385	0.381
SAH (%)	8 (12.5)	4 (8.0)	0.639	0.560	0.143–2.187	0.406
Percentage of cerebral volume lost (mean (SD))	41.72 (42.52)	26.60 (33.40)	0.187	−19.171§	11.562§	0.104§

sICH, symptomatic intracranial hemorrhage; SAH, subarachnoid hemorrhage; EVT, endovascular therapy; IVT, intravenous tPA; CI, confidence interval; ^§^Linear regression has been expressed as standard regression coefficient, standard error and *p* value.

In an unadjusted analysis of the full cohort, early neurological improvements were statistically more frequent in the EVT group (24, [48.0%] vs 17 [26.6%]; *p* = 0.03). On the overall series, similar results were found between the two groups on the excellent outcome (36 [56.2%], vs 27, [54.0%]; *p* = 0.960), functional independence (42 [65.6%], vs 37, [74.0%]; *p* = 0.449), mortality (13 [20.3%], vs 8, [16.0%]; *p* = 0.729), and sICH (6 [9.4%], vs 3, [6.0%]; *p* = 0.754).

In IPTW-adjusted analyses, EVT was not associated with a higher likelihood of a 3-month excellent outcome compared with IVT alone ([aOR], 1.575; 95%CI, 0.706–3.513; *p* = 0.269).

Sensitivity analysis excluding ten patients with multiple distal occlusions showed results in line with the previously reported ones (Tables S2–S4).

The results of univariable and multivariable analyses of factors associated with excellent outcomes are reported in Table 3.

**Table 3:** Perioperative univariable and multivariable analyses associated with excellent outcome

Variables	Univariable analysis		Multivariable analysis			
	No event (*N* = 51)	Excellent outcome (*N* = 63)	*p* Value	OR	95% CI	*p* Value
Female, *n* (%)	24 (47.1)	29 (46.0)	1.000			
Age, Y, median (IQR)	82.00 [77.00, 86.00]	78.00 [67.50, 82.00]	0.005	0.960	0.913–1.009	*0.106*
Weight, kg, median (IQR)	70.00 [65.00, 80.00]	72.00 [67.50, 78.00]	0.518			
Atrial fibrillation, *n* (%)	14 (27.5)	19 (30.2)	0.913			
Intrahospital stroke, *n* (%)	1 (2.0)	2 (3.2)	1.000			
Diabetes, *n* (%)	9 (17.6)	12 (19.0)	1.000			
Dyslipidemia, *n* (%)	14 (27.5)	15 (23.8)	0.820			
CAD, *n* (%)	17 (33.3)	12 (19.0)	0.127			
Cervical internal carotid artery stenosis, *n* (%)	10 (19.6)	7 (11.1)	0.316			
COPD, *n* (%)	3 (5.9)	3 (4.8)	1.000			
Previous stroke, *n* (%)	11 (21.6)	6 (9.5)	0.126			
Neoplasia, *n* (%)	2 (3.9)	2 (3.2)	1.000			
Dementia, *n* (%)	0 (0.0)	2 (3.2)	0.571			
Hypertension, *n* (%)	32 (62.7)	39 (61.9)	1.000			
Smoking habit, *n* (%)	5 (9.8)	7 (11.1)	1.000			
Chronic kidney disease, *n* (%)	3 (5.9)	2 (3.2)	0.809			
Home antiplatelet therapy, *n* (%)	27 (52.9)	20 (31.7)	0.036			
Home anticoagulant therapy, *n* (%)	6 (11.8)	5 (7.9)	0.712			
Statins, *n* (%)	7 (13.7)	13 (20.6)	0.473			
**mRS pre treatment,** ***n*** **(%)**			0.174			
0	39 (76.5)	55 (87.3)				
1	6 (11.8)	6 (9.5)				
2	6 (11.8)	2 (3.2)				
Atherosclerotic occlusion, *n* (%)	15 (29.4)	23 (36.5)	0.549			
**Menon score,** ***n*** **(%)**			0.031			
0	4 (7.8)	1 (1.6)				
1	1 (2.0)	0 (0.0)				
2	9 (17.6)	7 (11.1)				
3	16 (31.4)	11 (17.5)				
4	12 (23.5)	18 (28.6)				
5	9 (17.6)	26 (41.3)				
Intravenous tPA, *n* (%)	43 (84.3)	55 (87.3)	0.853			
EVT, *n* (%)	23 (45.1)	27 (42.9)	0.960			
Baseline NIHSS score (mean (SD))	12.08 (4.96)	7.54 (4.75)	<0.001	0.723	0.630–0.830	*<0.001*
Early neurological improvement, *n* (%)	9 (17.6)	32 (50.8)	0.001	20.251	4.790–85.639	*<0.001*
Time from onset to IVT, min, (mean (SD))	131.45 (77.57)	122.47 (86.16)	0.603			
Time from onset to groin puncture, min, (mean (SD))	225.43 (141.13)	258.92 (128.24)	0.389			
Time from onset to recanalization, min, (mean (SD))	266.70 (137.83)	306.23 (124.76)	0.297			
Procedure time, min, (mean (SD))	41.48 (18.24)	52.08 (42.44)	0.273			
ICH, *n* (%)	10 (19.6)	4 (6.3)	0.063			
sICH, *n* (%)	6 (11.8)	3 (4.8)	0.303			

CAD: coronary artery disease; COPD: chronic obstructive pulmonary disease; Y: year; kg: kilogram; ml: millilitres; min: minutes; IQR: interquartile range; SD: standard deviation; EVT, endovascular therapy; sICH, symptomatic intracranial hemorrhage; IVT, intravenous tPA; NA: not applicable.

In multivariable analysis, early neurological improvement (OR 20.251, 95% CI: 4.790–85.639, *p* < 0.001) was significantly associated with excellent outcomes. On the other hand, a lower baseline NIHSS score (OR 0.723, 95% CI: 0.630–0.830, *p* < 0.001) was significantly associated with excellent outcomes.

In IPTW-adjusted analyses, no significant difference was found between treatment groups for functional independence (aOR, 2.024; 95% CI, 0.845–4.848; *p* = 0.116), early neurological improvement (aOR, 2.218; 95% CI, 0.937–5.247; *p* = 0.073), and mortality (aOR, 0.498; 95% CI, 0.177–1.406; *p* = 0.191) at 3 months follow-up. Moreover, no significant difference was observed for sICH (aOR, 0.493; 95% CI, 0.102–2.385; *p* = 0.381) and SAH (aOR, 0.560; 95%CI, 0.143–2.187; *p* = 0.406) between treatment groups.

In patients whose data on pre-treatment CTP TMAX > 6 s and after-treatment FIV were available, a slightly higher percentage of cerebral volume lost in IVT group compared with EVT ± IVT group (41.72 ± 42.52% vs 26.6 ± 33.40%, *p* = 0.187) was found. After adjustment for propensity score weighting, a not statistically significant difference was confirmed (adjusted linear regression estimate, −19.171, *t* value, 11.562; *p* = 0.104).

## 4 Discussion

The Current American Heart Association (AHA)/American Stroke Association (ASA) guidelines provide a weak recommendation (class 2b) for EVT in patients with DMVO [27], while European guidelines do not give specific recommendations in this field [28].

Distal clots account for 25–40% of AISs and are associated with high rates of disability regardless of treatment [19]. Intravenous tPA is known to be more effective in smaller thrombus volumes of distal than in large clots of big vessels [29].

Although IVT alone is associated with a low reperfusion rate (around 50%) of distal occlusions [30], functional independence may be achieved in over 65% of the cases, with excellent outcomes obtained in 50% of the patients [31]. These results comply with the present study, which demonstrates that EVT ± IVT does not entail benefits in terms of functional independence, excellent outcome, early neurological improvement, and percentage of cerebral volume lost in patients with primary DMVO stroke when compared to IVT alone.

The main finding of the current study is that among patients with primary DMVO stroke, the treatment approach (EVT ± IVT vs IVT alone) did not influence the odds of functional independence, excellent outcome, early neurological improvement, and the percentage of cerebral volume lost.

Distal occlusions are burdened by long and tortuous courses, small artery calibers, and fragile arterial walls that may increase the likelihood of endovascular procedure-related complications [32]. Recently, progress in retriever and aspiration technologies has improved endovascular devices, making them able to remove distal clots [33].

In the absence of randomized clinical trials, evidence for EVT in DMVO is restricted to observational studies with promising results in terms of reperfusion rates, efficacy, and safety.

In this view, Saber et al. [34] showed higher rates of excellent outcomes in patients affected by DMVO of anterior circulation after EVT compared to BMT, without increased risks of mortality or sICH.

Similarly, in the PLATO study [35], EVT was associated with a greater likelihood of achieving an excellent outcome compared with BMT on PCA occlusions despite high rates of sICH and mortality.

Moreover, the TOPMOST registry suggested the potential benefit of EVT compared with BMT regarding early neurological improvement on PCA occlusions [36].

The same study group [37] found similar clinical outcomes comparing mechanical thrombectomy and BMT analysing primary isolated distal ACA occlusions.

The meta-analysis of Loh et al. [38] performed on 2,469 patients across 16 studies showed significantly better functional independence after EVT than BMT, but mechanical thrombectomy led to a higher rate of sICH in the mild strokes subgroup (NIHSS <6).

All cited studies [34,35,36,37,38] compared the efficacy and safety of EVT ± IVT with BMT in patients with primary DMVO stroke. However, the BMT group includes patients who were treated with supportive care without IVT in a percentage ranging from 15 to 60%. Hence, a selection bias in favour of the EVT group may be present.

To support this claim, other studies [39,40] comparing EVT ± IVT and IVT alone have failed to demonstrate the superiority of EVT, even if the interventional treatment was not burdened by increasing sICH and operative mortality. Another recent report [41] comparing reperfusion strategies found that EVT + IVT did not differ from IVT in terms of good functional status at 3-month follow-up.

Our results need to be confirmed. For this purpose, ongoing rCTs (DISTAL trial, the American DISTAL, Canadian ESCAPE-MeVO – NCT05151172 – and FRONTIER trial) may disclose interesting data on EVT for distal occlusions.

## 5 Limitations

Our study is limited by its nonrandomized retrospective and observational design. The EVT group presented higher NIHSS, longer time from onset to IVT, and more home anticoagulant therapy and could be at higher risk of developing worse post-procedural outcomes. Even if the doubly robust adjustment mitigates the baseline differences between groups, we cannot exclude the possibility of residual bias. The small sample size may affect statistical inference; therefore, rCTs are needed to confirm our results.

## 6 Conclusions

This study supports the hypothesis that patients with primary DMVO stroke treated with EVT (±IVT) or IVT alone have comparable outcomes. Given the benefit of vascular reperfusion, EVT could be the treatment choice when IVT is not suitable.

## Supplementary Material

Supplementary material
