# Supplementary material for: Endovascular thrombectomy versus intravenous thrombolysis for primary distal, medium vessel occlusion in acute ischemic stroke
Source: Open Med (Wars). 2024 May 13;19(1):20240966. doi: 10.1515/med-2024-0966 (PMC11097045; doi:10.1515/med-2024-0966)
Supplement: Supplementary material [file med-2024-0966-sm.pdf]

# Supplementary material

## S1 Methods

### S1.1 Identification of the study registries and primary contribution to the treatment arms

| Italian centre | EVT ± IVT cohort | IVT cohort | Total |
|---|---|---|---|
| Genova, San Martino Hospital | 37 | 33 | 70 |
| Roma, Gemelli Hospital | 13 | 31 | 44 |

EVT: Endovascular therapy; IVT: intravenous therapy.

### S1.2 Outcome definitions

Functional independence was defined as a mRS score 0–2 at 3 months.

Excellent outcome was defined as a mRS score 0–1 at 3 months.

Modified Rankin Score (mRS) is widely accepted as a 6 point disability scale with possible scores ranging from 0 to 5. A separate category of 6 is usually added for patients who expire [1].

Early neurologic improvement was specified as an improvement in NIHSS score at day 1 of at least 4 points or reaching 0 point [2].

Impact of treatment on the percentage of brain tissue lost was calculated as the ratio: Final infarction volume (FIV)/tissue at risk of infarction ($CTP_{TMAX}$ > 6s) ×100 [3].

FIV was calculated as the total hypodense dimensions area in each axial slice ipsilateral to DMVO and automatically summed to volumes using segmentation CT software[4].

Based on previous studies, $T_{\max}$ > 6s on CT perfusion was considered a reasonable estimate of tissue at risk of infarction in the absence of reperfusion [5].

Endovascular procedure related complications:

1) Subaracnoid haemorrhage or arterial perforation was defined as contrast material extravasation during endovascular treatment and/or blood suffusion in sub-arachnoid spaces at CT follow-up [6].
2) Embolization to new arterial territory/distal embolization in target territory was defined as a clot that during retrieval manoeuvres can migrate to a proximal previously unaffected territory or distally within the target artery. In distal embolization, the migrated clot can remain in the same vessel or break up and dissipate into many multiple tiny branches and possibly affect other surrounding vessel territories [6].
3) Arterial dissection appears as a localized contrast pocket or a double lumen or an intimal flap on angiography images affecting cervical segment of ICA and intracranial arteries. Other indirect indications of dissection may be arterial occlusion, stenosis, string sign, aneurysm, or pseudoaneurysm [6].
4) Symptomatic ICH (sICH) was defined according to European Cooperative Acute Stroke Study – 2 (ECASS II criteria). sICHwas diagnosed if the new observed ICH was associated with any of the following conditions: (1) NIHSS score increased >4 points than that immediately before worsening; (7) NIHSS score increased >2 points in 1 category; (4) deterioration led to intubation, hemicraniectomy, external ventricular drain placement, or any other major interventions. In addition, the symptom deteriorations could not be explained by causes other than the observed ICH [7].

### S1.3 DMVO definitions

Distal M2-segment of MCA stroke was defined as an occlusion of the M2 branches above the mid-height of the insula [8].

M2 MCA branch patterns include bifurcation, trifurcation, tetrafurcation, and candelabra. M2 MCA occlusions may occur in dominant segments that are similar to LVO or in non-dominant segments that are similar to M3. In this way non-dominant M2 stroke was defined as an occlusion of the smaller branch of MCA beyond the main bifurcation [8].

M3-segment stroke was defined as an occlusion of branch of MCA above the insula [8].

M4-segment stroke was defined as an occlusion of the branch of MCA when it exit the Sylvian fissure [8].

**Table S1:** Covariate balance analyses in weighted samples for the intravenous tPa vs endovascular therapy

| Variable | Type | Unadjusted sample | | | | | | | Adjusted sample | | | | | | |
|---|---|---|---|---|---|---|---|---|---|---|---|---|---|---|---|
| | | Intravenous rtPa 64 pts | | Endovascular therapy 50 pts | | Balance measures | | | Intravenous rtPa 56.94 pts | | Endovascular therapy 9.75 pts | | Balance Measures | | |
| | | Mean | Standard deviation | Mean | Standard deviation | Mean difference | Variance ratio | KS | Mean | Standard deviation | Mean | Standard deviation | Mean difference | Variance ratio | KS |
| Propensity score | Distance | 0.3793 | 0.1721 | 0.5144 | 0.1724 | 0.7843 | 1.0041 | 0.3581 | 0.4365 | 0.1810 | 0.4409 | 0.1823 | 0.0260 | 1.0145 | 0.0918 |
| Female | Binary | 0.5469 | 0.4978 | 0.3600 | 0.4800 | −0.3822 | . | 0.1869 | 0.4695 | 0.4991 | 0.4807 | 0.4996 | 0.0228 | . | 0.0111 |
| Age | Contin. | 77.5625 | 10.9427 | 76.3200 | 12.1226 | −0.1076 | 1.2273 | 0.1138 | 77.2624 | 11.6373 | 77.6171 | 11.2655 | 0.0307 | 0.9371 | 0.0563 |
| Atrial fibrillation | Binary | 0.2500 | 0.4330 | 0.3400 | 0.4737 | 0.1983 | — | 0.0900 | 0.2756 | 0.4468 | 0.2632 | 0.4404 | −0.0274 | . | 0.0124 |
| Diabetes | Binary | 0.2031 | 0.4023 | 0.1600 | 0.3666 | −0.1120 | — | 0.0431 | 0.1903 | 0.3925 | 0.1767 | 0.3814 | −0.0353 | . | 0.0136 |
| Dyslipidemia | Binary | 0.2656 | 0.4417 | 0.2400 | 0.4271 | −0.0590 | — | 0.0256 | 0.2391 | 0.4265 | 0.2265 | 0.4186 | −0.0291 | . | 0.0126 |
| CAD | Binary | 0.2500 | 0.4330 | 0.2600 | 0.4386 | 0.0229 | — | 0.0100 | 0.2790 | 0.4485 | 0.2858 | 0.4518 | 0.0157 | . | 0.0069 |
| Cervical internal carotid artery stenosis | Binary | 0.1250 | 0.3307 | 0.1800 | 0.3842 | 0.1534 | — | 0.0550 | 0.1531 | 0.3601 | 0.1574 | 0.3642 | 0.0120 | . | 0.0043 |
| Previous stroke | Binary | 0.1094 | 0.3121 | 0.2000 | 0.4000 | 0.2526 | — | 0.0906 | 0.1194 | 0.3242 | 0.1339 | 0.3406 | 0.0405 | . | 0.0145 |
| Hypertension | Binary | 0.6250 | 0.4841 | 0.6200 | 0.4854 | −0.0103 | — | 0.0050 | 0.6062 | 0.4886 | 0.6061 | 0.4886 | −0.0003 | . | 0.0001 |
| Smoking habit | Binary | 0.0938 | 0.2915 | 0.1200 | 0.3250 | 0.0850 | — | 0.0262 | 0.1200 | 0.3250 | 0.1132 | 0.3169 | −0.0220 | . | 0.0068 |
| Atherosclerotic occlusion | Binary | 0.3906 | 0.4879 | 0.2600 | 0.4386 | −0.2816 | — | 0.1306 | 0.3511 | 0.4773 | 0.3613 | 0.4804 | 0.0220 | . | 0.0102 |
| Baseline NIHSS score | Contin. | 8.4375 | 5.3980 | 11.0200 | 4.9218 | 0.5000 | 0.8313 | 0.2706 | 9.6816 | 5.8839 | 10.0190 | 4.7762 | 0.0653 | 0.6589 | 0.1093 |

**Table S2:** Sensitivity analysis: baseline characteristics and medical history excluding patients with multiple distal occlusions

| Variable | IVT group (59) | EVT ± IVT group (45) | *p* value |
|---|---|---|---|
| Female, no. (%) | 32 (54.2) | 16 (35.6) | 0.090 |
| Age, y, median (IQR) | 79.00 [74.00, 84.00] | 78.00 [72.00, 83.00] | 0.648 |
| Weight, kg, median (IQR) | 70.00 [67.00, 80.00] | 72.00 [66.00, 78.00] | 0.564 |
| Atrial fibrillation, no. (%) | 15 (25.4) | 17 (37.8) | 0.255 |
| Intrahospital stroke, no. (%) | 1 (1.7) | 2 (4.4) | 0.811 |
| Diabetes, no. (%) | 12 (20.3) | 7 (15.6) | 0.712 |
| Dyslipidemia, no. (%) | 16 (27.1) | 11 (24.4) | 0.934 |
| CAD, no. (%) | 16 (27.1) | 13 (28.9) | 1.000 |
| Cervical internal carotid artery stenosis, no. (%) | 8 (13.6) | 9 (20.0) | 0.540 |
| COPD, no. (%) | 4 (6.8) | 2 (4.4) | 0.935 |
| Previous stroke, no. (%) | 6 (10.2) | 10 (22.2) | 0.157 |
| Neoplasia, no. (%) | 1 (1.7) | 3 (6.7) | 0.429 |
| Dementia, no. (%) | 1 (1.7) | 1 (2.2) | 1.000 |
| Hypertension, no. (%) | 38 (64.4) | 27 (60.0) | 0.798 |
| Smoking habit, no. (%) | 6 (10.2) | 6 (13.3) | 0.849 |
| Chronic kidney disease, no. (%) | 2 (3.4) | 2 (4.4) | 1.000 |
| Home antiplatelet therapy, no. (%) | 27 (45.8) | 17 (37.8) | 0.538 |
| Home anticoagulant therapy, no. (%) | 1 (1.7) | 10 (22.2) | 0.002 |
| Statins, no. (%) | 10 (16.9) | 10 (22.2) | 0.671 |
| mRS pre treatment, no. (%) | | | 0.914 |
| 0 | 48 (81.4) | 38 (84.4) | 0.868 |
| 1 | 7 (11.9) | 5 (11.1) | |
| 2 | 4 (6.8) | 2 (4.4) | |
| Occlusion side, no. (%) | | | 1.000 |
| Right | 29 (47.5) | 21 (46.7) | |
| Left | 31 (52.5) | 24 (53.3) | |
| Atherosclerotic occlusion, no. (%) | 25 (39.1) | 13 (26.0) | 0.205 |
| Tissue at risk of infarction ($CTP_{tmax}$ > 6s), ml, mean (SD) | 50.08 (39.39) | 98.10 (107.72) | 0.134 |
| Occlusion site on CTA, no. (%) | | | 0.186 |
| Distal or non codominant M2-segment | 19 (27.5) | 26 (47.3) | |
| M3-segment | 32 (46.4) | 21 (38.2) | |
| M4-segment | 1 (1.5) | 0 (0) | |
| A2/A3-segment | | 6 (8.7) | 6 (10.9) |
| P2/P3-segment | 11 (15.9) | 2 (3.6) | |
| Menon score, no. (%) | | | 0.168 |
| 0 | 0 (0.0) | 1 (2.2) | |
| 1 | 1 (1.7) | 0 (0.0) | |
| 2 | 4 (6.8) | 9 (20.0) | |
| 3 | 13 (22.0) | 12 (26.7) | |
| 4 | 20 (33.9) | 9 (20.0) | |
| 5 | 21 (35.6) | 14 (31.1) | |

*(Continued)*

**Table S2:** *Continued*

| Variable | IVT group (59) | EVT ± IVT group (45) | *p* value |
|---|---|---|---|
| Intravenous tPA, no. (%) | 59 (100.0) | 31 (68.9) | <0.001 |
| Baseline NIHSS score (mean (SD)) | 7.88 (5.10) | 10.44 (4.72) | 0.010 |
| Time from onset to IVT, min, (mean (SD)) | 106.29 (71.07) | 157.21 (95.20) | 0.006 |
| Time from onset to groin puncture, min (mean (SD)) | NA | 245.39 (137.63) | NA |
| Time from onset to recanalization, min (mean (SD)) | NA | 290.82 (134.72) | NA |
| Procedure time, min (mean (SD)) | NA | 48.36 (34.73) | NA |

CAD: coronary artery disease; COPD: chronic obstructive pulmonary disease; Y: year; kg: kilogram; ml: millilitres; min: minutes; IQR: interquartile range; SD: Standard deviation; CTA: computed tomography angiogram; EVT, endovascular therapy; IVT, intravenous tPA; NA: not applicable.

**Table S3:** Sensitivity analysis: covariate balance analyses in weighted samples for the intravenous tPa vs endovascular therapy excluding patients with multiple distal occlusions

| Variable | Type | Unadjusted sample | | | | | | | Adjusted sample | | | | | | |
|---|---|---|---|---|---|---|---|---|---|---|---|---|---|---|---|
| | | Intravenous rtPa 59 pts | | Endovascular therapy 45 pts | | Balance measures | | | Intravenous rtPa 51.59 pts | | Endovascular therapy 34.87 pts | | Balance measures | | |
| | | Mean | Standard deviation | Mean | Standard deviation | Mean difference | Variance ratio | KS | Mean | Standard deviation | Mean | Standard deviation | Mean difference | Variance ratio | KS |
| Propensity score | Distance | 0.3620 | 0.1764 | 0.5254 | 0.1978 | 0.8721 | 1.2573 | 0.3913 | 0.4237 | 0.1904 | 0.4322 | 0.2037 | 0.0454 | 1.1445 | 0.1006 |
| Female | Binary | 0.5424 | 0.4982 | 0.3556 | 0.4787 | −0.3824 | . | 0.1868 | 0.4670 | 0.4989 | 0.4842 | 0.4997 | 0.0352 | — | 0.0172 |
| Age | Contin. | 76.7797 | 10.8645 | 75.8667 | 12.3133 | −0.0786 | 1.2845 | 0.1149 | 76.3830 | 11.1682 | 77.0452 | 11.4442 | 0.0570 | 1.0500 | 0.0713 |
| Atrial fibrillation | Binary | 0.2542 | 0.4354 | 0.3778 | 0.4848 | 0.2681 | — | 0.1235 | 0.2879 | 0.4528 | 0.2788 | 0.4484 | −0.0196 | — | 0.0090 |
| Diabetes | Binary | 0.2034 | 0.4025 | 0.1556 | 0.3624 | −0.1249 | — | 0.0478 | 0.1880 | 0.3907 | 0.1665 | 0.3726 | −0.0560 | — | 0.0214 |
| Dyslipidemia | Binary | 0.2712 | 0.4446 | 0.2444 | 0.4298 | −0.0612 | — | 0.0267 | 0.2422 | 0.4284 | 0.2337 | 0.4232 | −0.0195 | — | 0.0085 |
| CAD | Binary | 0.2712 | 0.4446 | 0.2889 | 0.4532 | 0.0394 | — | 0.0177 | 0.3190 | 0.4661 | 0.3213 | 0.4670 | 0.0052 | — | 0.0023 |
| Cervical internal carotid artery stenosis | Binary | 0.1356 | 0.3424 | 0.2000 | 0.4000 | 0.1730 | — | 0.0644 | 0.1703 | 0.3759 | 0.1747 | 0.3797 | 0.0119 | — | 0.0044 |
| Previous stroke | Binary | 0.1017 | 0.3022 | 0.2222 | 0.4157 | 0.3316 | — | 0.1205 | 0.1092 | 0.3118 | 0.1324 | 0.3389 | 0.0639 | — | 0.0232 |
| Hypertension | Binary | 0.6441 | 0.4788 | 0.6000 | 0.4899 | −0.0910 | — | 0.0441 | 0.6134 | 0.4870 | 0.6139 | 0.4868 | 0.0011 | — | 0.0006 |
| Smoking habit | Binary | 0.1017 | 0.3022 | 0.1333 | 0.3399 | 0.0984 | — | 0.0316 | 0.1289 | 0.3351 | 0.1278 | 0.3339 | −0.0032 | — | 0.0010 |
| Atherosclerotic occlusion | Binary | 0.4237 | 0.4941 | 0.2667 | 0.4422 | −0.3350 | — | 0.1571 | 0.3770 | 0.4846 | 0.3894 | 0.4876 | 0.0264 | — | 0.0124 |
| Baseline NIHSS score | Contin. | 7.8814 | 5.1027 | 10.4444 | 4.7221 | 0.5214 | 0.8564 | 0.2904 | 8.9468 | 5.5506 | 9.4563 | 4.5189 | 0.1037 | 0.6628 | 0.1180 |

**Table S4:** Sensitivity analysis: clinical outcomes and the doubly robust matching estimators for confounding adjustment for intravenous tPA vs endovascular therapy excluding patients with multiple distal occlusions

| Variables | Overall series | | | Doubly robust adjustment‡ | | |
|---|---|---|---|---|---|---|
| | IVT *N* = 59 pts | EVT ± IVT *N* = 45 pts | *p* value | Odds ratio | 95% CI | *p* value |
| Excellent outcome at 3 months (%) | 36 (61.0) | 27 (60.0) | 1.000 | 1.790 | 0.754–4.246 | 0.190 |
| Functional independence at 3 months (%) | 42 (71.2) | 37 (82.2) | 0.283 | 2.730 | 0.971–7.679 | 0.060 |
| Early neurological improvement (%) | 17 (28.8) | 24 (53.3) | 0.020 | 2.358 | 0.961–5.788 | 0.064 |
| Mortality (%) | 9 (15.3) | 5 (11.1) | 0.746 | 0.513 | 0.143–1.844 | 0.309 |
| sICH (%) | 6 (10.2) | 3 (6.7) | 0.781 | 0.452 | 0.090–2.263 | 0.336 |
| SAH (%) | 8 (13.6) | 4 (8.9) | 0.668 | 0.539 | 0.132–2.201 | 0.392 |
| Percentage of cerebral volume lost (mean (SD)) | 40.69 (43.80) | 27.09 (34.06) | 0.266 | −16.086§ | 12.473§ | 0.204§ |

sICH, symptomatic intracranial hemorrhage; SAH, subarachnoid hemorrhage; EVT, endovascular therapy; IVT, intravenous tPA; CI, confidence interval; §Linear regression has been expressed as standard regression coefficient, standard error and *p* value.

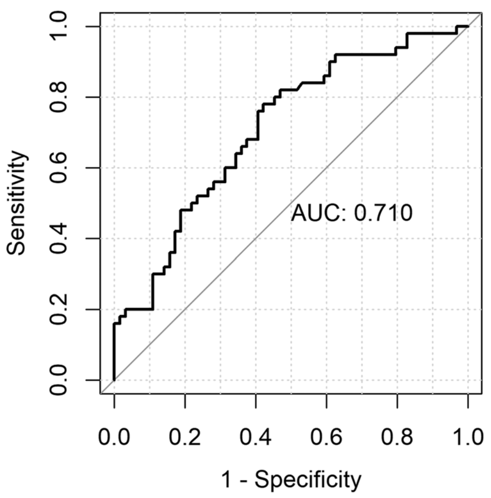


**Figure S1:** The C-statistics of the propensity score.

Distal anterior cerebral artery stroke was defined as an occlusion of branches above the anterior communicating artery [8].

Distal posterior cerebral artery stroke was defined as an occlusion of branches above the posterior communicating artery [8].

## References


[1] Banks JL, Marotta CA. Outcomes validity and reliability of the modified Rankin scale: implications for stroke clinical trials: a literature review and synthesis. Stroke. 2007 Mar;38(3):1091–6. doi: 10.1161/01.STR.0000258355.23810.c6

[2] Kobeissi H, Ghozy S, Bilgin C, Kadirvel R, Kallmes DF. Early neurological improvement as a predictor of outcomes after endovascular thrombectomy for stroke: a systematic review and meta-analysis. J Neurointerv Surg. 2023 Jun;15(6):547–51. doi: 10.1136/neurintsurg-2022-019008

[3] Suomalainen OP, Elseoud AA, Martinez-Majander N, Tiainen M, Forss N, Curtze S. Comparison of automated infarct core volume measures between non-contrast computed tomography and perfusion imaging in acute stroke code patients evaluated for potential endovascular treatment. J Neurol Sci. 2021 Jul;426:117483. doi: 10.1016/j.jns.2021.117483

[4] Strength of association between infarct volume and clinical outcome depends on the magnitude of infarct size: results from the ESCAPE-NA1 Trial. AJNR Am J Neuroradiol. 2021 Aug;42(8):1375–9. doi: 10.3174/ajnr.A7183

[5] Bouslama M, Ravindran K, Harston G, Rodrigues GM, Pisani L, Haussen DC, et al. Noncontrast computed tomography e-stroke infarct volume is similar to RAPID computed tomography perfusion in estimating postreperfusion infarct volumes. Stroke. 2021 Jan;52(2):634–41. doi: 10.1161/STROKEAHA.120.031651

[6] Salsano G, Pracucci G, Mavilio N, Saia V, Poggio MB, Malfatto L, et al. Complications of mechanical thrombectomy for acute ischemic stroke: Incidence, risk factors, and clinical relevance in the Italian Registry of Endovascular Treatment in acute stroke. Int J Stroke. 2021 Oct;16(7):818–27. doi: 10.1177/1747493020976681

[7] Hao Y, Yang D, Wang H, Zi W, Zhang M, Geng Y, et al. For the ACTUAL investigators (Endovascular treatment for acute anterior circulation ischemic stroke registry). Predictors for symptomatic intracranial hemorrhage after endovascular treatment of acute ischemic stroke. Stroke. 2017;48:1203–9. doi: 10.1161/STROKEAHA.116.016368

[8] Saver JL, Chapot R, Agid R, Hassan A, Jadhav AP, Liebeskind DS, et al. Thrombectomy for distal, medium vessel occlusions: a consensus statement on present knowledge and promising directions. Stroke. 2020;51:2872–84. doi: 10.1161/STROKEAHA.120.028956
